# Three-Dimensional Numerical Simulation of the Performance and Transport Phenomena of Oxygen Evolution Reactions in a Proton Exchange Membrane Water Electrolyzer

**DOI:** 10.3390/ma16031310

**Published:** 2023-02-03

**Authors:** Jinsong Zheng, Zhenye Kang, Bo Han, Jingke Mo

**Affiliations:** 1 Department of Aeronautics and Astronautics, Fudan University, Shanghai 200433, China; 2 School of Chemical Engineering and Technology, Hainan University, Haikou 570228, China; 3 Department of Aeronautics and Astronautics, Zhejiang University, Hangzhou 310027, China

**Keywords:** PEM water electrolysis, electrochemical performance, mass transfer, flow field patterns, three-dimensional model

## Abstract

Proton exchange membrane (PEM) water electrolysis, which is one of methods of hydrogen production with the most potential, has attracted more attention due to its energy conversion and storage potential. In this paper, a steady state, three-dimensional mathematical model coupled with the electrochemical and mass transfer physical fields for a PEM water electrolyzer was established. The influence of the different operation parameters on the cell performance was discussed. Moreover, the different patterns of the flow field, such as parallel, serpentine, multi-serpentine, and interdigitate flow fields, were simulated to reveal their influence on the mass transfer and current distribution and how they consequently affected the cell performance. The results of the numerical modeling were in good agreement with the experimental data. The results demonstrated that a higher temperature led to a better mass transfer, current distribution, and cell performance. By comparing the polarization curve, current, velocity, and pressure distribution, the results also indicated that the PEM water electrolyzer with a parallel flow field had the best performance. The results in this study can help in optimizing the design of PEM water electrolyzers.

## 1. Introduction

Hydrogen is considered to be a clean energy source and a promising replacement for traditional fossil fuel energy [1,2,3]. Proton exchange membrane (PEM) water electrolyzers are a type of device used in the production of hydrogen with the most potential [4,5,6]. Compared with other hydrogen production devices, PEM water electrolyzers have several advantages including a high purity of hydrogen production, high efficiency, compact structure, etc. [7,8,9].

The performance of PEM water electrolyzers is affected by many factors, including operation parameters such as temperature, pressure, reactant flow rate, internal structure parameters such as the thickness and the pore size of the liquid/gas diffusion layer, different flow fields, catalyst layer properties, and morphology [10,11,12]. In order to better understand the mechanism of electrochemical and mass transfer in PEM water electrolyzers and improve their performance, mathematical modeling and simulation can be used as a promising way to theoretically conduct investigations with less time and cost requirements, and this can then be used to optimize the design and improve the performance and efficiency.

Over the past few decades, most researchers have mainly focused on thermodynamic methods. Choi et al. [13] established a 1D steady-state model of a PEM water electrolyzer. The cell voltage was composed of Nernst potential, activation overpotential, ohmic overpotential and concentration overpotential. An equivalent electrical circuit analogy was provided for the sequential kinetic and transport resistances. They found that the reduction kinetics at the cathode were relatively fast, while the anodic overpotential was mainly responsible for the voltage drop. Onda et al. [14,15] conducted a 2D model simulation of a single cell and predicted the stack performance. The convection heat, conduction heat, heat transfer, latent heat of water evaporation, the absorbed heat by the entropy change, and the generated heat by the overpotential for the water electrolyzing reaction were discussed in detail. The shunt resistance was considered in the ohmic loss for the first time. The influence of different temperatures and pressures on the cell performance was compared and measured by experimentation. Gorgun et al. [16] first established a dynamic model under one-dimensional adiabatic conditions. They calculated the partial pressure of the substances at both ends of the anode and cathode through the molar conservation and ideal gas equation and then discussed the phenomena of the electro-osmotic drag force and the diffusion of water in a membrane electrode. The model was established by Simulink, and the polarization curve, efficiency, partial pressure, and current transient change were discussed. Marangio et al. [17] established a one-dimensional non-adiabatic steady-state model. From the perspective of the polarization curve, they studied the activation overpotential, concentration overpotential, and ohmic overpotential of the cathode and anode, respectively. The influence of mass transfer phenomenon on the activation/concentration overpotential and the influence of electrode and flow field plate shunt resistance on the ohmic overpotential were taken into account.

In previous studies, most models have used CFD software with the finite volume method to analyze the flow phenomena in the flow field and porous media. Nie et al. [18] established a 3D dynamic model of the flow field of a PEM water electrolyzer. Through the fluid control equation, the generation rate and the distribution of hydrogen/oxygen in the flow field were calculated by FLUENT at a certain water flow rate. Lobato et al. [19] studied the influence of different flow fields and established a 3D model, including three kinds of flow fields. The pressure drop, velocity distribution, and oxygen mole fraction distribution of the different flow fields were calculated.

Recent years, in order to establish a more accurate model, more researchers have tried to solve the questions of multi-physical field coupling. Kaya et al. [20] developed a 2D isothermal model to investigate the performance of a PEM water electrolyzer, and the effects of Pt and Pt–Ir anode catalysts were compared at the same conditions under different temperatures, membrane thicknesses, current collector lengths, and molar fraction distributions in the channel. Sezgin et al. [21] developed a 3D steady-state isothermal model to observe the effects of various parameters, inlet velocities of the gases, and conductivities of the polymer membrane, and the simulation results of the concentration, current density, and polarization curve were compared with experimental results. Haghayegh et al. [22] established a 3D isothermal steady-state model, coupled the mass transfer phenomenon in the flow field and porous media through the fluid control equation, and calculated the reaction kinetics in a membrane electrode, and they discussed the concentration distribution, pressure distribution, and the influence of temperature and pressure on the current density. Zhang et al. [23] established a 3D steady-state model. The mass transfer, heat transfer, and current distribution were coupled to figure out the influence of co-flow and counter-flow regimes and the influence of different widths of the flow field on the cell performance.

In this study, a steady state, three-dimensional model of a PEM water electrolyzer was established by coupling the electrochemical model, the mass conservation model and the hydrodynamics model. Hence, the current distribution, pressure distribution, transport of species and fluid dynamics were coupled by the electrochemical and mass transfer physical field. The performance of the PEM water electrolyzer under different working conditions were discussed, including the current density, temperature, and pressure. Different patterns of the flow field were simulated, and the performances of the cells using these flow fields were compared. The results can help in optimizing the design of PEM water electrolyzers.

## 2. Materials and Methods

**Figure 1:**
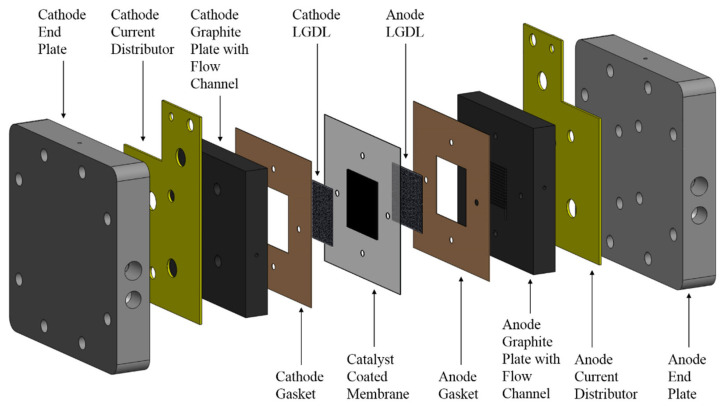
Schematic diagram of a standard PEM water electrolyzer.

The structure of a typical PEM water electrolyzer is shown in Figure 1. The polymer electrolyte membrane is in the middle of cell, which plays the role of conducting protons, and it can also isolate the connection between the reactants and products on both sides, ensuring the purity of the products. The catalyst layer is the reaction site in the PEM water electrolyzer, at which water is decomposed into hydrogen and oxygen. The catalyst layer is placed at both ends of the proton exchange membrane by ultrasonic spraying or other technologies, which are the anode catalyst layer and cathode catalyst layer, respectively. This combined structure is called the catalyst coating membrane (CCM). In order to transfer the reactants and products, a layer with a porous structure, entitled the diffusion layer, needs to be added outside the anode and cathode, respectively. The combined structure of the above CCM and the diffusion layer is collectively referred to as the membrane electrode assembly (MEA). Next to the MEA, from the inside to the outside, there are flow field plates, electrified plates, and end plates. The flow field plate is mainly used to control the internal flow state, and the electrified plate is used to connect with an external power supply to apply current to the PEM water electrolyzer. The outermost end plate plays a role of fixing the components.

The MEA is the core component of a PEM water electrolyzer. In addition to the role of the diffusion layer of transferring reactants, the CCM plays a catalytic role in the water electrolysis. The concept of a three-phase boundary (TPB) exists in the water electrolysis reaction [24,25,26]. It is believed that in the water electrolysis reaction, the oxygen evolution reaction (OER) and hydrogen evolution reaction (HER) only occur in a limited-space area, which is at the junction of the PEM, the catalyst, and the pores of the diffusion layer. Among them, the reaction kinetics of the OER have a great impact on the performance of water electrolysis.

The reaction in a PEM water electrolyzer can be expressed as follows:

(1) $$\mathrm{Anode}:\;H_{2}O\to 2H^{+}+2e^{-}+\frac{1}{2}O_{2}$$

(2) $$\mathrm{Cathode}:\;2H^{+}+2e^{-}\to H_{2}$$

(3) $$\mathrm{Total}:\;H_{2}O\to \frac{1}{2}O_{2}+H_{2}$$

**Figure 2:**
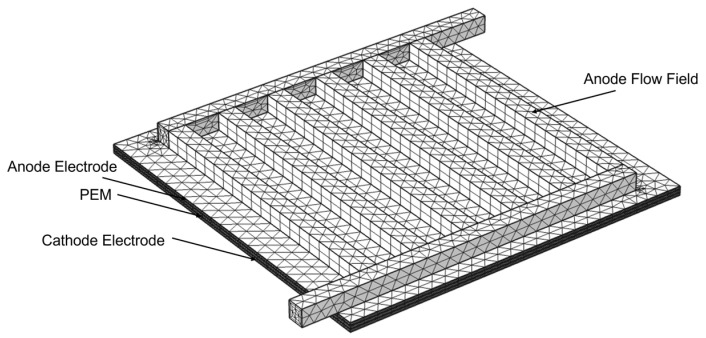
The computational domain of the model.

A flow field plate is also very important for the water electrolysis reaction [27]. In the process of water electrolysis, the reactant (water) and the product (hydrogen) exist in the anode simultaneously. The flow characteristics, diffusion phenomena, mass transfer, and heat transfer characteristics of the mixture play a decisive role in the concentration polarization, affecting the performance of the PEM water electrolyzer. At the same time, the flow field plate and the electrified plate also act the part of conducting electricity, ensuring the continuous progress of the electrochemical reaction. The grooves in the flow field plate are called flow channels. The main types of flow fields are shown in Figure 2, including serpentine flow fields, parallel flow fields, multi-channel serpentine flow fields, interdigital flow fields, grid flow fields, spiral flow fields, etc. The reactant (water) produces an oxygen and hydrogen flow in the diffusion layer and the flow field. According to the principle of PEM water electrolyzers, the supply and flow of water will affect its electrolysis efficiency after reaching the CCM, and different types of CCM will also affect the electrolysis efficiency, affecting the degree of electrochemical polarization. At the same time, the amount of products obtained by electrolysis at the cathode and anode and the difficulty of discharging the products from the diffusion layer and the flow field will cause a change in the substance concentration in the PEM water electrolyzer, which will have a certain impact on the concentration polarization. Under the combined action of electrochemistry and concentration polarization, PEM water electrolyzers with different flow fields and MEAs will have different performances, so it is necessary to study the types and properties of different structures.

### 2.1. Electrochemical Analysis

The PEM water electrolysis reaction is affected by the electrochemical reaction kinetics and the transport of protons and electrons. Among them, the mass transfer resistance in the flow field and the diffusion layer of the PEM hydrolysis cell have a very important influence on the above physical and chemical processes. The mass transfer resistance is mainly divided into the diffusion resistance of the reactant products, electro-osmotic drag resistance, and cross resistance [21,23,28]. Diffusion resistance is the overvoltage caused by the change in the equivalent resistance caused by the change in the concentration of the cathode and anode, and electro-osmotic drag resistance is the resistance caused by protons passing through the membrane, which is mainly related to the humidity of the PEM. Cross resistance is caused by the gas passing through the PEM, which has little impact. The mass transfer resistance will greatly affect the electrochemical reaction process and produce a certain degree of overpotential, affecting the performance of the PEM water electrolyzer.

By establishing a numerical model of the electrochemical reactions and mass transfer processes in a PEM water electrolyzer, the performance of the PEM water electrolyzer and the distribution of the physical quantities such as speed, pressure, material concentration, and current density distribution inside the PEM water electrolyzer can be simulated, which can give a more intuitive and clear understanding of the internal situation of the water electrolytic cell and also ensure the correctness of the performance comparison under different working conditions [29]. In this section, some assumptions about the mathematical model in this study are made, and then a complete water electrolysis model is established in three parts, namely an electrochemical model, a mass conservation model, and a momentum conservation model. Finally, the boundary conditions are set.

An electrochemical model was developed for a PEM water electrolyzer. A PEM water electrolyzer needs to consume external electric energy to split water. The polarization curve, which is the relationship between the current density and cell voltage, depicts the performance of a water electrolyzer. The cell voltage mainly consist of four parts, which can be expressed as follow [30]:

(4) $$E_{cell}=E_{OCV}+\eta_{act}+\eta_{ohm}+\eta_{diff}$$

where $E_{OCV}$ is the open circuit voltage, $\eta_{act}$ is the activation overpotential, $\eta_{ohm}$ is the ohmic overpotential, and $\eta_{diff}$ is the diffusion overpotential. The theoretical energy is the required energy for water splitting without any losses, which is represented by the open circuit voltage. The open circuit voltage is the reversible potential, which can be determined by the Nernst equation and can be calculated as follows [31]:

(5) $$E_{OCV}=-\frac{\Delta G}{nF}$$

where ΔG is the Gibbs free energy, n is the number of electrons transferred in the semi-reaction, and F is the Faraday constant.

During the electrochemical reaction in water splitting, the Gibbs free energy is affected by the temperature and pressure conditions. The difference in the behavior between a real gas and an ideal gas is dependent only on the pressure and the temperature and not on the presence of any other gases. At a given temperature, the “effective” pressure of a gas is given by its fugacity. By historical convention, fugacities have the dimension of pressure, so the dimensionless activity is given by:

(6) $$\alpha_{i}=\frac{f_{i}}{p^{*}}=\varphi_{i}y_{i}\frac{p}{p^{*}}$$

where $\varphi_{i}$ is the dimensionless fugacity coefficient of the species, $y_{i}$ is its mole fraction in the gaseous mixture (y=1 for a pure gas), and p is the total pressure. The value $p^{*}$ is the standard pressure; it may be equal to 1 atm (101.325 kPa) or 1 bar (100 kPa). The Gibbs free energy at a constant temperature and pressure can be calculated by the Nernst equation:

(7) $$\Delta G=\Delta G^{*}+RT\left[\ln\left(\frac{p_{H_{2}}p_{O_{2}}^{1/2}}{\alpha_{H_{2}O}}\right)\right]$$

where $\Delta G^{*}$ is the reference Gibbs free energy at standard pressure and different cell temperatures. The species enthalpies and entropies are used to calculate the reference Gibbs free energy as follows [32]:

(8) $$\Delta G^{*}=\Delta H^{*}-T\Delta S^{*}=\sum \nu_{i}H_{i}\left(T\right)-T\sum \nu_{i}S_{i}\left(T,P\right)$$

where $\nu_{i}$ is the stoichiometric coefficients.

The steady form of the charge balance can be expressed as:

(9) $$\nabla \cdot j_{l}+\nabla \cdot j_{s}=0$$

where $j_{l}$ is the ion current in the membrane, and $j_{s}$ is the electron current in the electrodes, both of which can be calculated by Ohm’s law:

(10) $$\nabla \cdot j_{l}=-\nabla \cdot \left(\sigma_{l}^{\mathrm{eff}}\nabla \phi_{l}\right)$$

(11) $$\nabla \cdot j_{s}=-\nabla \cdot \left(\sigma_{s}^{\mathrm{eff}}\nabla \phi_{s}\right)$$

where ∇ is the Laplace operator, $\sigma_{l}^{\mathrm{eff}}$ is the effective ion conductivity, and $\sigma_{s}^{\mathrm{eff}}$ is the effective conductivity of the electrodes, determined by the Bruggeman correction:

(12) $$\sigma_{l}^{\mathrm{eff}}=\sigma_{l}\varepsilon_{l}^{3/2}$$

(13) $$\sigma_{s}^{\mathrm{eff}}=\sigma_{s}\varepsilon_{s}^{3/2}$$

where $\sigma_{l}$ is the membrane ion conductivity coefficient, and $\varepsilon_{l}$ is the porosity. This can be calculated as follows [33]:

(14) $$\sigma_{l}=\left[0.005193\lambda -0.00326\right]\exp\left[1268\left(\frac{1}{303}-\frac{1}{T}\right)\right]$$

where λ is the humidification degree.

The activation and diffusion overpotentials at the anode and cathode catalyst layer depend on the electrode potential, electrolyte potential, and open circuit voltage, which can be expressed as follows:

(15) $$\eta_{act}+\eta_{diff}=\phi_{s}-\phi_{l}-E_{OCV}=\left(E_{cell}-\eta_{ohm}\right)-E_{OCV}$$

The activation overpotential is the energy loss caused by the electrochemical reaction, which can be calculated by the Butler-Volmer equation as follows [34]:

(16) $$j_{v}=a_{v}j_{0}\left[\exp\left(\frac{\alpha_{a}F}{RT}\eta_{act}\right)-\exp\left(-\frac{\alpha_{c}F}{RT}\eta_{act}\right)\right]$$

where $a_{v}$ is the electrode specific surface area, $\alpha_{a}$ and $\alpha_{c}$ are the charge transfer coefficients on the anode and cathode, respectively, R is the gas constant, and T is the cell temperature. $j_{0}$ is the exchange current density, which influences the diffusion overpotential, and it can be calculated as follows [35]:

(17) j0=j0,ref∏icici,refαavin∏icici,ref−αcvin

where the $c_{i}$ is the species concentration, and $c_{i,ref}$ is the reference concentration.

### 2.2. Momentum Conservation

The momentum equation in a porous domain is described by the Brinkman equation, in which the gas velocity is approximated by Darcy’s law and the continuity equation, which can be calculated as follows [36]:

(18) $$\frac{\rho }{\varepsilon }\left(\left(u\cdot \nabla \right)\frac{u}{\varepsilon }\right)=\nabla \cdot \left[-p+\left(\frac{\mu }{\varepsilon }\left(\nabla u\right)\right)-\frac{2\mu }{3\varepsilon }\left(\nabla \cdot u\right)\right]-\left(\frac{\mu }{k}+Q\right)u$$

(19) $$\nabla \cdot \left(\rho u\right)=Q$$

where ρ is the density of the gas mixture, ε is the electrode porosity, u is the mass velocity, μ is the viscosity, k is the electrode permeability, and Q represents the mass source, which can be calculated as:

(20) $$Q=\underset{m}{\sum }\underset{i}{\sum }R_{i,m}M_{i}$$

The Navier–Stokes and continuity equations without mass creation depict the flow in the gas channels, which can be expressed as follows [37]:

(21) $$Ρ\left(u\cdot \nabla \right)u=\nabla \cdot \left[-p+\left(\mu \left(\nabla u\right)\right)-\frac{2}{3}\mu \left(\nabla \cdot u\right)\right]$$

(22) $$\nabla \cdot \left(\rho u\right)=0$$

### 2.3. Mass Conservation

The flow distribution in the gas channel can be described by the Maxwell–Stefan equation because of the multicomponent diffusion and convection conditions [20]:

(23) $$\frac{\partial }{\partial t}\rho \omega_{i}+\nabla \cdot \left[\begin{array}{c} -\rho \omega_{i}\overset{N}{\underset{j=1}{\sum }}D_{ij}\left\{\frac{M}{M_{j}}\left(\nabla \omega_{j}+\omega_{j}\frac{\nabla M}{M}\right)+\left(x_{j}-\omega_{j}\right)\frac{\nabla P}{P}\right\}+\omega_{i}\rho u \end{array}\right]=q_{i}.\;$$

where $\omega_{i}$ is the mass fraction, P is the partial pressure, M is the molar mass, and $q_{i}$ is the species flux, which is a function of the local current density.

(24) $$q_{H_{2}}=\frac{j_{c}}{2F}M_{H_{2}}$$

(25) $$q_{O_{2}}=\frac{j_{a}}{4F}M_{O_{2}}$$

(26) $$q_{H_{2}O}=\frac{j_{a}}{2F}M_{H_{2}O}$$

The diffusivity in the free space can be expressed as follows [38]:

(27) $$D_{ij}=\frac{a{\left(\frac{T}{\sqrt{T_{ci}T_{cj}}}\right)}^{b}{\left(P_{ci}P_{cj}\right)}^{1/3}{\left(T_{ci}T_{cj}\right)}^{5/12}{\left(\frac{1}{M_{i}}+\frac{1}{M_{j}}\right)}^{1/2}}{P}$$

The effective diffusion coefficient in the electrodes, which is approximated by the Bruggeman correction, can be expressed as:

(28) $$D_{i,j}^{\mathrm{eff}}=D_{i,j}\varepsilon^{1.5}$$

The density of the gas mixture can be calculated as follows:

(29) $$\rho =\frac{\left(p+p_{\mathrm{ref}}\right)M}{\mathrm{RT}}$$

(30) $$M={\left(\sum_{i}\frac{w_{i}}{M_{i}}\right)}^{-1}$$

$x_{k}$ is the molar fraction, which can be calculated as:

(31) $$x_{k}=\frac{w_{k}}{M_{k}}M_{n}$$

In this research, the water inlet was related to the stoichiometry, and the mass flow rate can be expressed as follow:

(32) $$M_{flux_{in}}=\lambda M_{H2O}\frac{jA}{2F}$$

The electrochemical model was established by using the Nernst equation, the Butler–Volmer formula, Ohm’s law, and some semi-empirical formulas, and the mass conservation model was established by using the mass conservation equation, the Stefan–Maxwell formula, and some basic thermodynamic formulas. The hydrodynamic model was established by using the kinetic conservation and the Brinkman equation. By coupling the above three models, a complete mathematical model of a PEM water electrolyzer was established. The parameters and boundary conditions of the model were set on the basis of actual working conditions.

### 2.4. Physical modeling

The geometrical model used in this paper is shown in Figure 2. The structure from top to bottom was mainly composed of four parts: the anode flow field, which was composed of 1 mm × 1 mm microchannels; the anode electrode, including the anode gas diffusion layer and the anode catalyst layer; the PEM; and the cathode electrode, including the cathode gas diffusion layer and the cathode catalyst layer.

**Figure 3:**
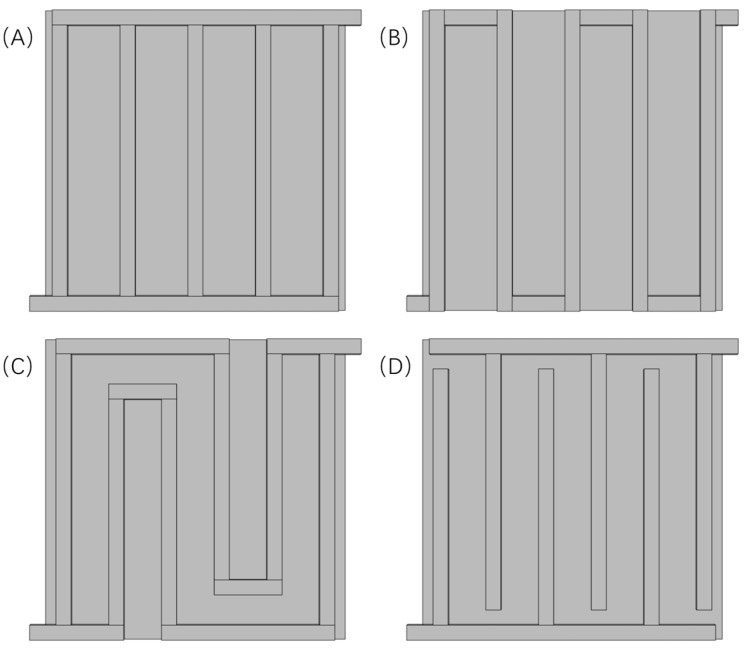
The different flow fields of PEMEC. (**A**) Parallel, (**B**) serpentine, (**C**) multi-serpentine, and (**D**) interdigitate.

In addition to the parallel flow field, the other three common flow fields were also numerically investigated in this paper. The different flow fields are as show in Figure 3.

By using COMSOL Multiphysics, the three-dimensional geometric model of a PEM water electrolyzer was established. FEM was used to mesh the geometric model and verify the grid quality. The mathematical model was substituted by region. The parameters were changed according to different experimental conditions, and the accuracy of the model was verified. The results showed that the mathematical model was in good agreement with the experimental results and had a high accuracy.

Geometric models of PEM water electrolyzers with different flow patterns were established and substituted into the same mathematical model.

## 3. Results and Discussions

### 3.1. Model Validation

The mathematical model in this study was established and calculated by using COMSOL Multiphysics. To validate the present model, the numerical results were compared with the previous experimental results reported by Hansen et al. [39] at the same working conditions and with a similar geometric form. The working conditions included a cell temperature of 403 K and a pressure of 1 atm, and the geometric parameters and other working conditions are shown in Table 1.

**Figure 4:**
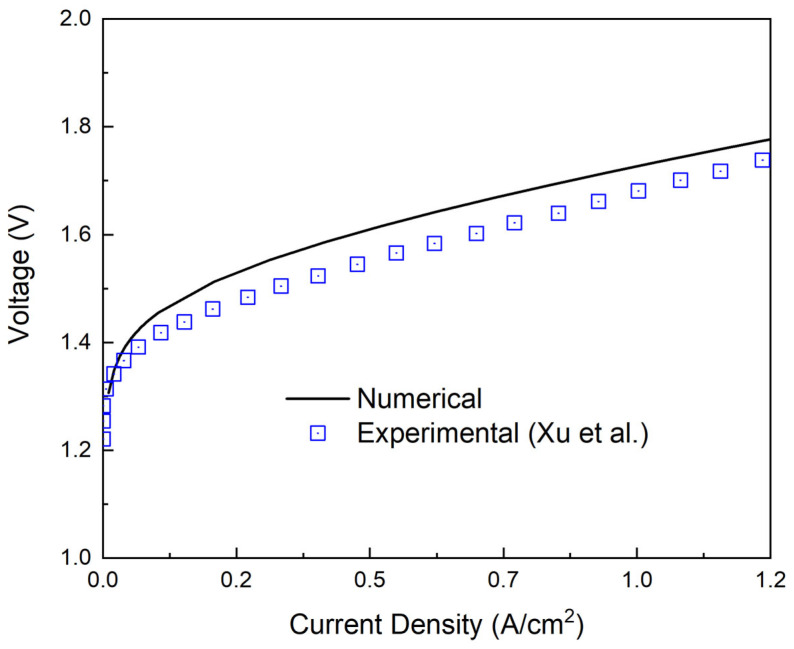
Comparison and validation of the present model with experimental data.

Figure 4 illustrates the comparison of the present results with the experimental data, and the results showed a good agreement with the experimental data. The coefficient of determination of fitting was 0.97. After the validation, the effective model was used to calculate and analyze the impact of different working conditions and geometric forms on the cell performance.

### 3.2. Effect of Different Current Density on Mass Transport

The hydrogen and oxygen production rates were closely related to the current density. According to Faraday’s law, the molar production rate ${\dot{n}}_{i}$ corresponded to the current density, which can be expressed as follows [30]:

(33) $${\dot{n}}_{i}=\frac{jA}{nF}$$

**Figure 5:**
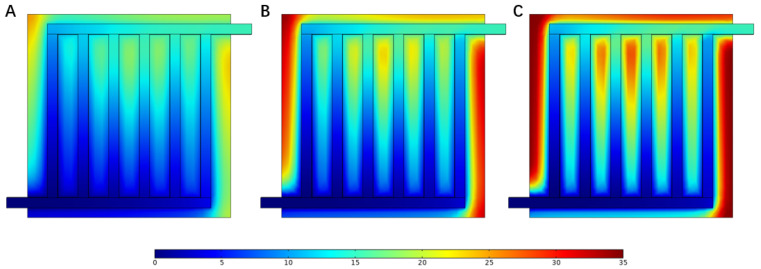
Oxygen mole fraction distribution in the anode under different current densities: (**A**)$\;0.5\;A\;{\mathrm{cm}}^{-2}$, (**B**) $1.0\;A\;{\mathrm{cm}}^{-2}$, and (**C**) $1.5\;A\;{\mathrm{cm}}^{-2}$.

The mole concentration was proportional to the molar production rate, which can be calculated by Equation (32). Figure 5 illustrates the oxygen mole concentration distribution at the anode at different current densities. It can be seen that when the current density was increased, the oxygen mole concentration increased in the anode, while most of the increase was at the edge of electrode. It is worth mentioning that when the current density was too high, the oxygen generated at the electrode was hard to remove, which slowed the continuous progress of the reaction.

**Figure 6:**
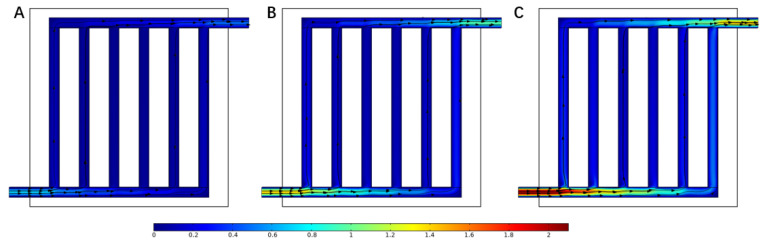
Velocity distribution in the flow field under different current densities: (**A**)$\;0.5\;A\;{\mathrm{cm}}^{-2}$, (**B**) $1.0\;A\;{\mathrm{cm}}^{-2}$, and (**C**) $1.5\;A\;{\mathrm{cm}}^{-2}$.

According to Equation (30), the current density was related to the inlet mass flow rate, which influenced the velocity in the flow field. Figure 6 illustrates the velocity field at the same current density as above. At the inlet of the flow field, the velocity at $1.5\;A\;{\mathrm{cm}}^{-2}$ was $2.1{\;m\;s}^{-1}$, which was three times larger than the velocity at $0.5\;A\;{\mathrm{cm}}^{-2}$ and twice the velocity at $1.0\;A\;{\mathrm{cm}}^{-2}$. This can also explain the increase in the molar concentration.

### 3.3. Effect of Cell Temperature

**Figure 7:**
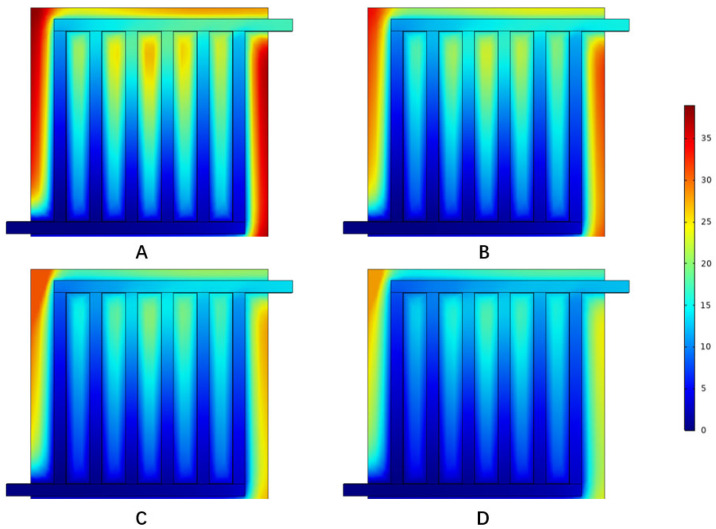
Oxygen mole fraction distribution under different temperatures: (**A**) 40 °C, (**B**) 80 °C, (**C**) 120 °C, and (**D**) 160 °C.

From the present model, it was found that the open circuit voltage, activation overpotential, mass concentration, diffusion coefficient, and membrane conductivity were closely related to the cell temperature. Figure 7 illustrates the oxygen mole concentration distribution at different temperatures. When the temperature increased, the molar concentration dropped evidently at the edge of electrode, and concentration drop was small at the boundary of the electrode and the flow field.

### 3.4. Effect of Different Flow Fields

**Figure 8:**
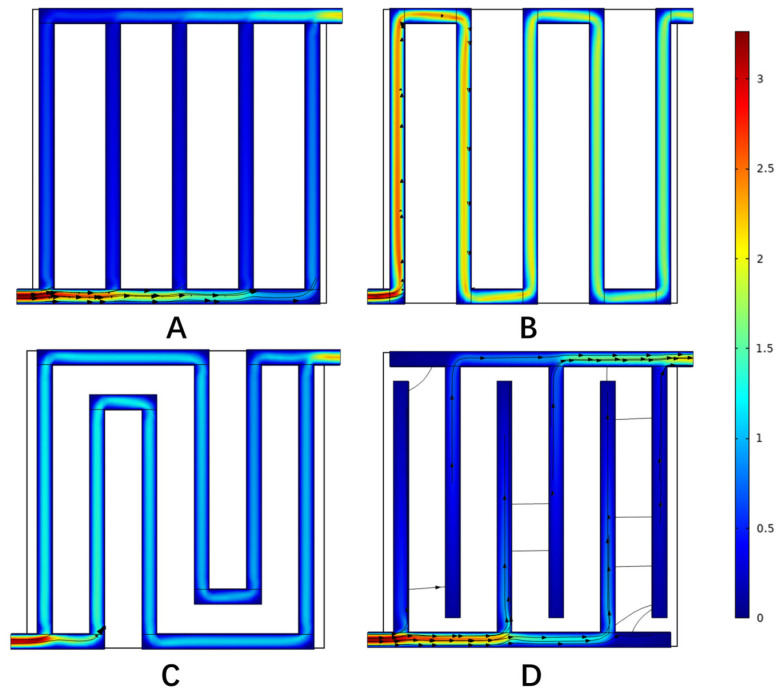
Velocity distribution in microchannels with different flow fields. (**A**) Parallel, (**B**) serpentine, (**C**) multi-serpentine, and (**D**) interdigitate.

Figure 8 shows the velocity distribution in different flow fields when the current density was $1.0\;A\;{\mathrm{cm}}^{-2}$. It can be seen that at the channel turning point in the flow field, the velocity decreased along the channel, which was caused by the higher pressure difference between the adjacent channels compared to other areas. Comparing the velocity distribution of each flow field, the maximum velocity in the parallel flow field was $1.32\;{m\;s}^{-1}$, the maximum velocity in the single-channel serpentine flow field was $1.62\;{m\;s}^{-1}$, the maximum velocity in the multi-channel serpentine flow field was $1.56\;{m\;s}^{-1}$, and the maximum velocity in the interdigital flow field was $1.36\;{m\;s}^{-1}$. The velocity distribution in the two serpentine flow fields was relatively uniform. The peak velocity of the parallel flow field and interdigital flow field was mainly at the inlet and outlet, and the velocity in the flow channel was relatively small, which led to a slower migration of the reactants through the porous media. In this way, although the reaction could be carried out more fully, the products were discharged slowly.

**Figure 9:**
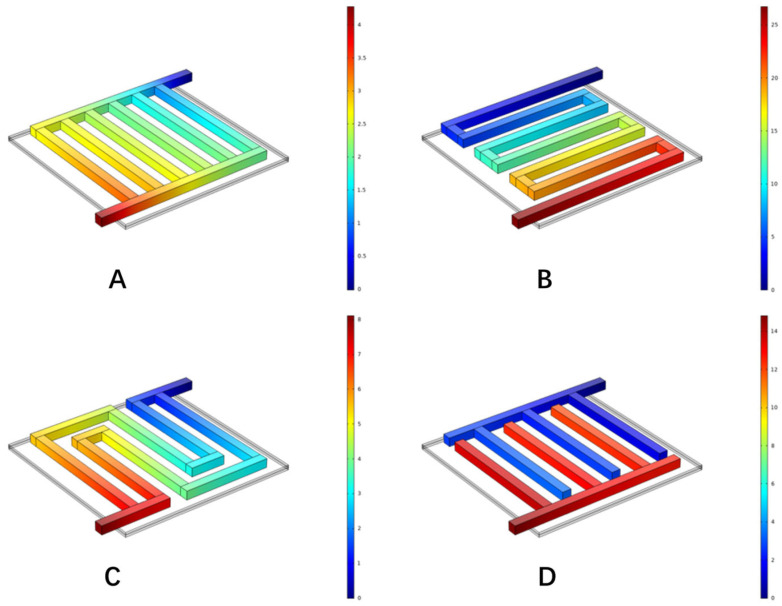
Pressure distribution in microchannels with different flow fields. (**A**) Parallel, (**B**) serpentine, (**C**) multi-serpentine, and (**D**) interdigitate.

The anode pressure distribution of hydro digesters in different flow fields under a current density of $1.0\;A\;{\mathrm{cm}}^{-2}$ is shown in Figure 9. The pressure distribution map shows which flow field had a low pressure drop. A PEM water electrolyzer with a low-pressure-drop flow field can consume less energy when distributing the reactants, thus improving the performance to a certain extent. In the figure, the pressure drop of the parallel flow field was 3.46 Pa, and the pressure dropped uniformly along the axis connecting the inlet and outlet. The pressure drop of the single serpentine flow field was 38.9 Pa, and that of the multi serpentine flow field was 11.6 Pa. The pressure of both serpentine flow fields decreased gradually along the direction of the flow passage. The pressure drop of the interdigital flow field was 7.08 Pa, and the pressure of the inlet side and outlet side of the flow channel decreased slowly along their respective flow channels, while the pressure of the flow channel from the inlet to the outlet dropped rapidly. It can be clearly seen that the pressure drop of the parallel flow field was lower than that of the other flow fields. By comparing the two serpentine flow fields, it can be seen that there were fewer bends in the channel of the parallel flow field. Based on the analysis of the velocity distribution in the previous section, the flow velocity in the parallel flow field was also relatively slow, so the flow was less obstructed. Compared with the interdigital flow field, the main reason for this is that the mixed gas can be directly discharged to the outlet, while the interdigital flow field must pass through the porous media, which increases the pressure drop.

The oxygen concentration distribution is the result of the interaction between the oxygen evolution rate and the mixture flow. On the one hand, a higher oxygen concentration can reflect a faster overall reaction rate, and the same current density indicates more reaction points, and the electrochemical polarization is weak, so it has a better electrolytic performance. On the other hand, this may indicate that the slower flow rate affects the discharge of oxygen, and a stronger concentration polarization, a worse electrolytic performance, and an uneven distribution of the oxygen region also verifies this point. By comparing the oxygen concentration with the current density distribution, the point which dominates the reaction can be obtained.

**Figure 10:**
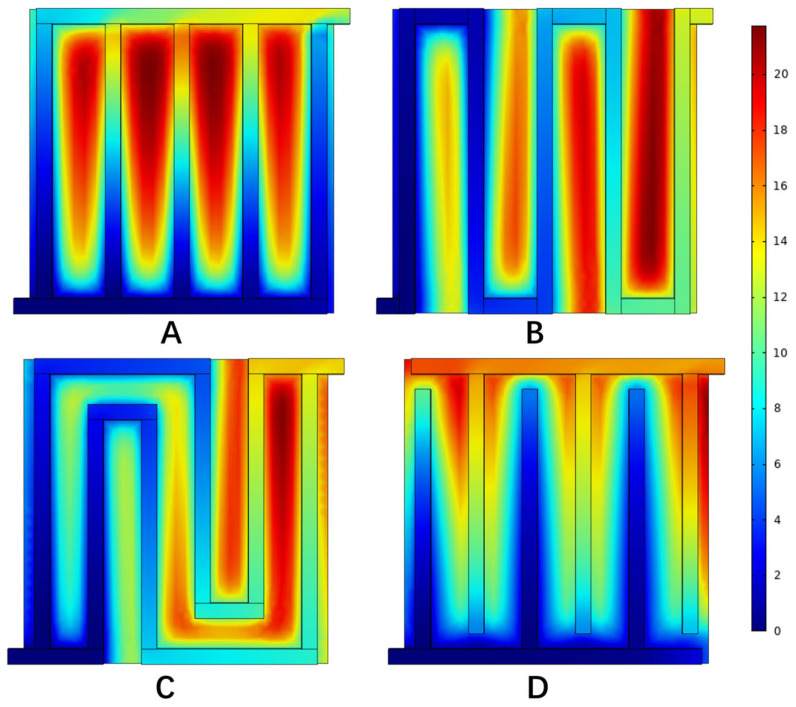
Oxygen mole concentration in anode electrode with different flow fields. (**A**) Parallel, (**B**) serpentine, (**C**) multi-serpentine, and (**D**) interdigitate.

Figure 10 shows the anodic oxygen concentration distribution in anode electrode of the PEM water electrolyzer with different flow fields when the current density was $1.0\;A\;{\mathrm{cm}}^{-2}$. The oxygen concentration in each flow field increased from the inlet to the outlet. Among them, the maximum oxygen concentration of the anode electrode of the PEMEC with a parallel flow field reached $13.7\;{\mathrm{mol}\;m}^{-3}$, which was located in the middle of the side near the outlet, indicating that the water entered the porous medium for a full reaction and had the highest oxygen generation rate on the whole. The maximum oxygen concentration of the interdigital flow field reached $13.5\;{\mathrm{mol}\;m}^{-3}$, which was mainly located at the end of interdigital flow field on the inlet side and at the edge of electrode. At the same time, it can be seen that the parallel and interdigital flow fields had a high oxygen concentration due to the slow flow rate and the accumulation of products. The maximum oxygen concentration of the two serpentine flow fields reached $11.0\;{\mathrm{mol}\;m}^{-3}$ and increased uniformly from the inlet to the outlet.

**Figure 11:**
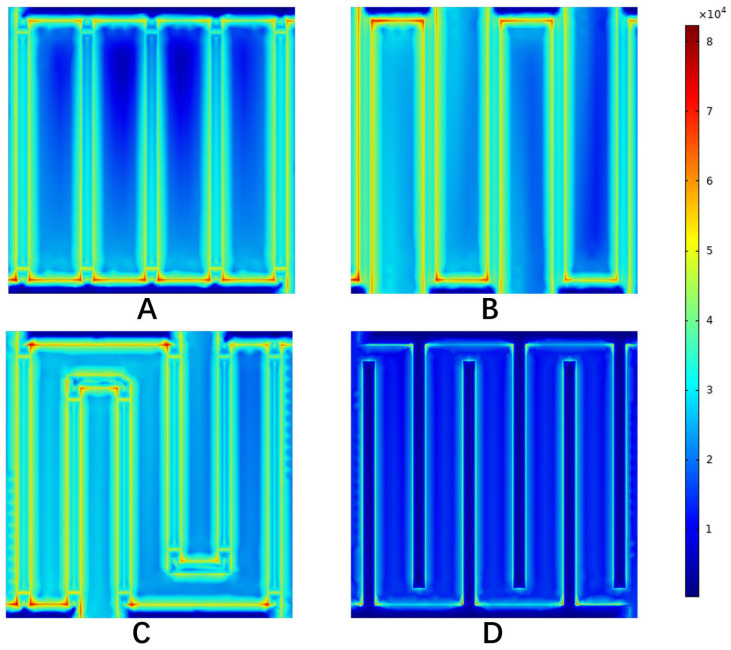
The current density distribution on anode electrode under different flow fields. (**A**) Parallel, (**B**) serpentine, (**C**) multi-serpentine, (**D**) interdigitate.

As the reactants entered the flow field and penetrated into the electrode, they were continuously consumed, resulting in a concentration difference. In the flow process, pressure gradients appeared in the middle and edge of the flow channel. Figure 11 shows the corresponding current density distribution, with an average current density of $1.0\;A\;{\mathrm{cm}}^{-2}$. It can be seen from the figure that the water electrolysis reaction was mainly distributed at the junction of the flow field and the electrode. The maximum current density of the anode in the parallel flow field was $1.87\;A\;{\mathrm{cm}}^{-2}$, and the maximum current density was mainly at the junction of the inlet and outlet section and the flow channel. The maximum current density of the single-channel serpentine flow field was $1.76\;A{\;\mathrm{cm}}^{-2}$, and that of the multi-channel serpentine flow field was $1.8\;A\;{\mathrm{cm}}^{-2}$. The maximum current density was mainly at the position where the flow channel bends. The current density distribution of the above three flow fields was relatively uniform. The highest current density of the interdigital flow field anode was $2.1\;A\;{\mathrm{cm}}^{-2}$. The highest current density was mainly at the junction of the inlet and outlet sections and the flow channel. At the same time, the reaction points were concentrated at the junction of the flow field and the electrode, which seriously affected the reaction efficiency. In combination with the oxygen concentration distribution, it can be considered that the high oxygen concentration position of the interdigital flow field was due to the accumulation of oxygen that was not easily discharged. To sum up, the reasons for the performance differences between the different flow fields could be analyzed from the perspective of the oxygen concentration distribution and current density distribution.

## 4. Conclusions

In this research, a steady state, three-dimensional mathematical model coupled with electrochemical and mass transfer physical fields for a PEM water electrolyzer was established and validated. Under the same working condition, the flow velocity, oxygen and water concentration distribution, current density distribution, and polarization curve of the different models were compared. The results showed that the single-channel and multi-channel serpentine flow fields had faster flow velocities than the parallel and interdigital flow fields. Although the distribution of the reactants and products was more uniform, they caused a greater pressure drop, so the diffusion overpotential increased. The oxygen concentration distribution of the two serpentine flow fields was similar, and the parallel flow field and interdigital flow field had slightly higher values. However, the interdigital flow field was mainly caused by the difficulty of the mixture entering and exiting the porous media, the low oxygen generation rate, and the difficulty in discharging. Taking the polarization curve as the main factor and considering many aspects comprehensively, the parallel flow field had a good performance in terms of electrolyzing water.

## Figures and Tables

**Table 1:** Basic parameters used in the PEMEC modeling.

Description, Symbol	Value, Unit
MEA active area, A	$4{\;\mathrm{cm}}^{2}$
Operating pressure, P	1, 2, 4 atm
Operating temperature, T	40, 80, 120, 160 °C
Electrode porosity, ε	0.4
Electrode specific surface area, $a_{v}$	$10^{9}\;{\mathrm{cm}}^{2}$
Anode charge transfer coefficients, $\alpha_{a}$	2
Cathode charge transfer coefficients, $\alpha_{c}$	0.5
Anode exchange current density, $\alpha_{0}{}_{a}$	$6\times 10^{-10}\;A\;{\mathrm{cm}}^{-2}$
Cathode exchange current density, $\alpha_{0}{}_{a}$	$0.34\;A\;{\mathrm{cm}}^{-2}$
PEM	$Nafion^{@}\;115$
Electrode thickness, δ	200 mm

## Data Availability

Not applicable.
